# An update: the emerging evidence of complement involvement in COVID-19

**DOI:** 10.1007/s00430-021-00704-7

**Published:** 2021-04-03

**Authors:** Qin Li, Zi Chen

**Affiliations:** 1 Guangzhou Municipal Research Institute of Clinical Medicine, The Second Affiliated Hospital of South China University of Technology, Guangzhou, Guangdong China; 2 Department of Respiratory and Critical Care Medicine, The First Affiliated Hospital, Nanjing Medical University, 300 Guangzhou Road, Nanjing, 210029 Jiangsu China

**Keywords:** COVID-19, SARS-CoV-2, Complement, Complement inhibitor

## Abstract

The current outbreak of coronavirus disease 2019 (COVID-19) has affected people around the world. Typically, COVID-19 originates in the lung, but lately it can extend to other organs and lead to tissue injury and multiorgan failure in severe patients, such as acute respiratory distress syndrome (ARDS), kidney failure and sepsis or systemic inflammation. Given that COVID-19 has been detected in a range of other organs, the COVID-19-associated disease is an alert of aberrant activation of host immune response which drives un-controlled inflammation that affects multiple organs. Complement is a vital component of innate immunity where it forms the first line of defense against potentially harmful microbes, but its role in COVID-19 is still not clear. Notably, the abnormal activation and continuous deposits of complement components were identified in the pre-clinical samples from COVID-19 patients, which have been confirmed in animal models. Recent evidence has revealed that the administration of complement inhibitors leads to relieve inflammatory response in ARDS. Hence, we speculate that the targeting complement system could be a potential treatment option for organ damage in COVID-19 patients.

## Introduction

The outbreak of coronavirus disease 2019 (COVID-19) caused by Severe Acute Respiratory Syndrome Coronavirus-2 (SARS-CoV-2) occurred in December of 2019 and rapidly spread worldwide, resulting in a global pandemic of unprecedented magnitude. Great efforts are underway in the clinical and scientific communities to find effective ways to stop the spread of this novel virus [1–4].

SARS-CoV-2 belongs to the family of Coronaviridae, seven of which are known to give rise to human disease [5]. During past years, two endemic outbreaks, SARS-CoV in 2003 and Middle East Respiratory Syndrome (MERS)-CoV in 2012 [6], occurred with significant consequences for public health. Compared to the antecedent epidemics of SARS-CoV and MERS-CoV, the transmission rate of SARS-CoV-2 is strikingly rapid since even asymptomatic individuals can transmit the virus. Patients with SARS-CoV-2 infection present a wide range of clinical presentations varies from asymptomatic to life-threatening that require ICU support [2, 3, 7, 8]. Patients who become critically ill have a risk of acute respiratory distress syndrome (ARDS), thrombotic complications, and multiorgan failure. This cataclysmic pattern of immune activation response, together with complement products found in clinical samples as documented here, are highly suggestive of aberrant activation of complement system in COVID-19.

**Fig. 1:**
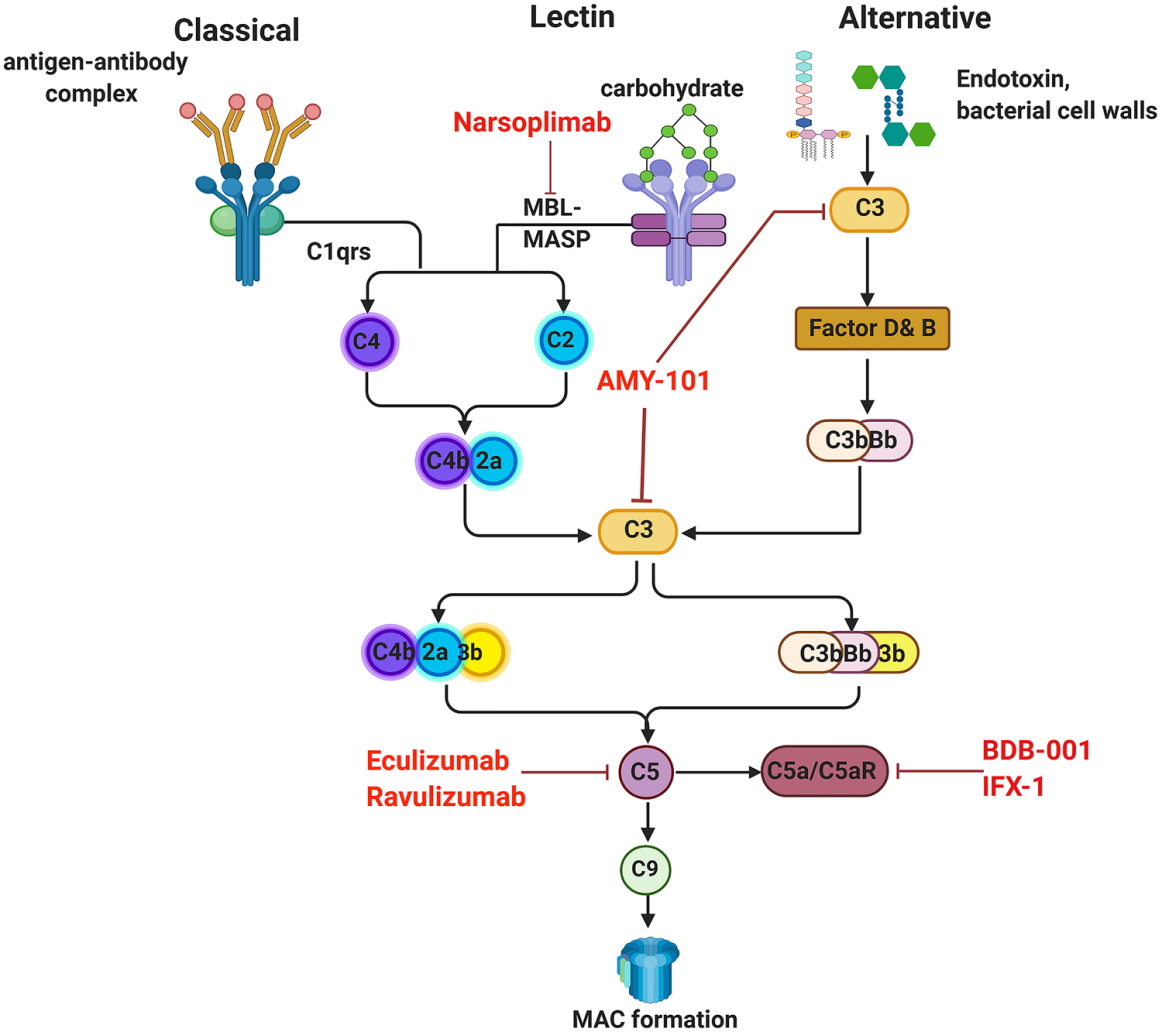
Complement pathways and complement inhibitors currently applied in clinical trials for COVID-19

The complement system acts at the front line of host defense system to pathogens. It is part of the innate immune system and composed of nearly 30 more proteins [9]. The complement system can be activated in three traditional pathways (Fig. 1): the classical pathway (CP, initiated by antigen–antibody complex on pathogen surfaces), the lectin pathway (LP, binding of mannan-binding lectin (MBL) or ficolin to mannose containing carbohydrates on the surface of bacteria or viruses), and the alternative pathway (AP, spontaneously activated complement components binding to pathogen surface), characterized by proteolytic cascade events fueled by the C3 and C5 convertases. Eventually, the cleavage product C5b initiates the final step of complement activation, assembly of the membrane attack complex (MAC) C5b-9.

Although complement functions predominantly as innate immune response against bacterial or viral infections, unrestrained complement activation may promote detrimental inflammatory reaction and ultimately lead to tissue damage. For instance, the complement activation product, C5a, is a potent anaphylatoxin that involves in exacerbating inflammatory reactions and participates in multiorgan failure during sepsis [10]. In addition, there is an established crosstalk between the coagulation and fibrinolysis through thrombin- and plasmin-mediated complement activation. The C3a and C5 cleavage products, C5a and C5b, as well as mannose-associated serine protease 2 (MASP-2), influence many aspects of coagulation [11]. Recent evidence has revealed the complement system has well-described roles in influenza virus and respiratory syncytial virus infection. The most recent review article indicated the activation of complement and contact system may play a role in coronavirus pathogenesis by mice model study. However, further study is still needed to confirm the suspicions [12].

This review will focus on the recent literature and detail the significant emerging clinical and experimental findings of complement involvement in SARS-CoV-2, highlighting therapeutic strategies against complement.

## Clinical evidence supporting complement engagement in COVID-19

### Circulation pool (red blood cells/plasma/peripheral blood mononuclear cells/sera)

Despite a high infection rate of COVID-19, only a small proportion of patients require intensive care. No reliable clinical biomarkers are available yet to identify these patients early in their disease course. The circulation pool can provide convenient and accessible biological material for biomarker discovery and profiling. Thus, we start from the circulation pool to review complement activation in COVID-19 patients, including red blood cells (RBCs), plasma, peripheral blood cells (PBMCs), and sera, which might be useful for potential peripheral biomarker discovery (Table 1).

**Table 1:** Collective pre-clinical evidence of complement involvement in the circulation pool

Circulation pool	Collective evidence of complement deposition	References
Serum	A general upregulation of complement system proteins, including MAC proteins such as C5, C6, and C8	Shen et al*.* [13]
Serum/plasma	Consistent activation of both the classical complement pathway (C1R, C1S, and C8A) as well as the alternative pathway factor B (CFB) and the complement modulators: factor I (CFI) and H (CFH)	Messner et al*.* [14]
Plasma	Plasma levels of sC5b-9 and C5a as markers of complement activation in COVID-19 patients	Peffault de Latour et al*.* [20] and Cugon et al*.* [21]
PBMCs	COVID-19 patients showed the up-regulated genes enriched in “complement activation”	Xiong et al*.* [15]
RBCs	IgG bound to RBCs from most hospitalized patients with COVID-19 was observed in most of the DAT tests	Berzuini et al*.* [16] and Hendrickson et al*.* [17]
	RBCs from patients with COVID-19 not only bound C3b/iC3b/C3dg and C4, but also bound viral spike protein	Lam et al*.* [19]

High-throughput transcriptomic and proteomic data are useful to draw pictures of host immune response against SARS-CoV-2 infection and these data may yield potential critical diagnostic markers or therapeutic targets for monitoring COVID-19 patients. Two independent groups from Europe and China applied mass spectrometry to analyze the proteome of serum/plasma from COVID-19 patients [13, 14]. A general upregulation of complement system proteins, including the classical complement pathway (C1R, C1S, and C8A), the alternative pathway factor B, the complement modulators factors I and factor H, as well as MAC proteins such as C5, C6, and C8, were reflected in COVID-19 patients [13, 14]. Additionally, transcriptional changes in PBMCs from COVID-19 patients also showed that the up-regulated genes are enriched in “complement activation”, “humoral immune response mediated by circulating immunoglobulin”, and “B cell mediated immunity”, suggesting that activated complement components may play an important role in the disease process of COVID-19 [15].

In a recent study, Berzuini et al*.* described IgG bound to RBCs from most hospitalized patients with COVID-19 as observed in most of the positive direct antiglobulin test (DAT), with eluates reactive with RBCs from other patients with SARS-CoV-2 but not with reagent RBCs. They also observed C3d expression in 12% of the positive DATs, either in combination with IgG or in isolation [16, 17]. The classical pathway is primarily activated by antibodies bound to viral antigens. The formed immune complexes interact with C1q and initiate the classical pathway, which is associated with C3b deposition with viral envelop [18]. Similarly, another study reported that RBCs from patients with COVID-19 not only bound C3b/iC3b/C3dg and C4, but also bound viral spike protein, suggesting that viral infection may activate the classical pathway of the complement system [19].

A study from Saint-Louis hospital in France documented complement activity in plasma from 103 patients with COVID-19 [20]. In this study, the authors found that the levels of C3 and C4 were increased in some patients, and the level of circulating sC5b-9 was increased in 64% of the patients, highlighting the systemic complement activation during COVID-19 infection [20]. Additionally, the levels of sC5b-9 were significantly higher in patients than the healthy donors, and higher in the patients with severe disease than in the moderate disease group [20]. These findings were consistent with a single-center case series study from Italy [21].

Collectively, these data indicate that complement is activated in the circulation of COVID-19 patients, and it could cause further damage of microvasculature of different organs through systemic activated complement-mediated cytotoxicity.

### Lung

The respiratory system is primarily involved in COVID-19, and a surge of patients developed ARDS. Clinical evidence suggests that activation of the complement system is critical in the pathogenesis of COVID-19-related lung injury (Table 2). A recent preprint study reported that the paraformaldehyde-fixed lung tissue from patients who died of COVID-19 revealed strong expression of complement components MBL, MASP-2, C4a, C3, and the terminal MAC C5b-9, suggesting LP complement hyper-activation occurred in the lungs of COVID-19 patients. In this article, the researchers demonstrated that viral N proteins from SARS-Cov, MERS-Cov, and SARS-Cov-2 could bind MBL and activate MASP-2, leading to the unrestrained activation of the complement cascade that participates in the pathogenesis of coronavirus infection [22, 23]. Simultaneously, a report from five severe COVID-19 infection cases documented significant deposits of terminal complement components C5b-9, C4d, and MBL-MASPs in the microvasculature in the lung [24]. This study also found a co-localization of COVID-19 spike glycoprotein with C4d and C5b-9 in the inter-alveolar septa [24]. As we all known, the main reason for admission and mortality of COVID-19 patients is respiratory failure. A recent study showed that the complement system markers of sC5b-9, C5a, C3bc, C3bBbP, and C4d were consistently increased in hospitalized COVID-19. And especially, MAC sC5b-9 was highly correlated with systemic inflammation and respiratory failure [25].

**Table 2:** Clinical evidence of complement deposition in multi-organs from COVID-19 patients

Organ	Collective evidence of complement activation	References
Lung	Strong expression of MBL, MSAP2, C4a, C3, and MAC C5b-9 in paraformaldehyde-fixed lung tissue from patients who died of COVID-19 N proteins from SARS-CoV-2 could bind MBL and activate MASP-2	Gao et al. [23]
	Striking deposition of C5b-9, C4d, and MASP2 in the microvasculature were found in the lung A co-localization of COVID-19 spike glycoprotein with C4d and C5b-9 were also found in the inter-alveolar septa	Magro et al*.* [24]
	Transcriptome data from lung tissue of two patients with SARS-CoV-2 infection, and in vitro respiratory epithelial cells showed the complement pathway was one of the most significant induced pathways	Yan et al*.* [26]
Kidney	C5b-9 deposition on tubules in all six cases along with low expression of C5b-9 on glomeruli and capillaries in kidney from COVID-19 patients	Diao et al. [30]
Skin	Three patients with critical COVID-19 had purpuric skin rash with striking deposition of C5b-9 and C4d in both grossly involved and normally appearing skin, and colocalization of SARS-CoV-2-specific spike glycoprotein	Magro et al*.* [24]
	Two children with Kawasaki-like hyperinflammatory syndrome were reported as a novel SARS-CoV-2 induced phenotype in Italy, with obvious complement consumption	Licciardi et al*.* [31]

Further evidence comes from RNA-seq data from lung tissues of two patients with SARS-CoV-2 infection. When comparing the transcriptome of patients to controls, the researchers found 36 canonical pathways significantly be induced in patients and 5 of the enriched pathways (14%) were annotated as complement pathways. To confirm if these are the SARS-Cov2-induced lung cell-intrinsic complement, the authors checked the transcriptome of primary human bronchial epithelial cells, A549 and angiotensin-converting enzyme 2 (ACE2)-transduced A549 cells when infected with SARS-Cov-2 in vitro. Results showed that the complement pathways were among the most highly enriched of all pathways following SARS-Cov-2 infection, one of which was the most significantly induced pathway in A549-ACE2 cells [26]. These results suggest that respiratory epithelial cells are a primary source of complement within lung tissues.

### Kidney

The main SARS-Cov-2 receptor, ACE2, is highly expressed in the apical brush borders of the proximal tubules and podocytes with less intensity, highlighting the relevance of kidney as a target of SARS-Cov-2 [27]. SARS-Cov-2 may directly bind with ACE2 expressed on kidney cells and lead to immune responses and the complement cascade locally [28]. Recent evidence has shown that a considerable incidence of renal abnormalities are associated with COVID-19, including proteinuria, hematuria and acute kidney injury [29]. The pathophysiology of COVID-19-associated renal injury is not well understood yet. A recent online report was done in kidney tissues from postmortems in China [30] (Table 2). The authors found C5b-9 depositions on tubules in all six cases along with low expression of C5b-9 on glomeruli and capillaries in two cases, indicating SARS-Cov-2 infection induced complement activation occurred in the kidney as well [30]. A preliminary autoptic examination of kidneys from seven COVID-19 patients in Italy was consistent with data regarding these Chinese patients [28]. These results indicate that the local complement activation and inflammation responses may contribute partially to kidney damage and dysfunction.

### Skin

Cutaneous manifestations have been described in the setting of COVID-19 (Table 2). The role of systemic complement activation and microvascular thrombosis has been implicated in pathogenic mechanism of skin lesion. A report from Magro et al*.* documented that three patients with critical COVID-19 had purpuric skin rash with striking deposition of C5b-9 and C4d in both grossly involved and normally appearing skin, and colocalization of SARS-CoV-2-specific spike glycoprotein [24]. Two children with Kawasaki-like hyperinflammatory syndrome were reported as a novel SARS-CoV-2 induced phenotype in Italy [31] with other reports following. In that study, the authors described signs of mucocutaneous involvement, including conjunctivitis, fissured lips, skin rash, erythema, and edema of the hands and feet, developed in both patients [31]. Blood tests revealed elevated makers of inflammation, lymphopenia, thrombocytopenia, and complement consumption [31]. It has been speculated that these manifestations might be a result of complement associated microvascular injury and thrombosis.

## The in vivo evidence of complement activation in animal models during coronavirus infection

Complement activation has been previously recognized as an important factor in the pathogenesis of SARS and MERS, both diseases mediated by coronaviruses that are similar to SARS-Cov-2. Thus, we will now review the scientific literature on animal models that were studied for the relationship between these coronavirus infections and complement (Table 3).

**Table 3:** The study of complement activation in mice models during coronavirus infection

Genotype	Collective evidence of complement activation	References
WT	Complement is activated after SARS-CoV MA15 (the mouse-adapted SARS-CoV) infection as demonstrated by elevated C3 activation products (C3 fragments C3a, C3b, iC3b, C3dg, and C3c)	Gralinski et al*.* [32]
C3^−/−^	C3^−/−^ mice showed a partial reduction of respiratory dysfunction, pathology, immune cell infiltration, and cytokine responses in the lung, compared with WT mice	Gralinski et al*.* [32]
B^−/−^	Factor B^−/−^ mice had less weight loss than WT mice	Gralinski et al*.* [32]
C4^−/−^	C4^−/−^ mice had less weight loss than WT mice	Gralinski et al. [32]

In wild-type mice, complement is activated after SARS-CoV MA15 (the mouse-adapted SARS-CoV) infection as demonstrated by elevated C3 activation products (C3 fragments C3a, C3b, iC3b, C3dg, and C3c) [32]. These mice exhibited pronounced lung pathology in parallel with humans, including inflammatory cells in the large airway and parenchyma, perivascular cuffing, thickening of the interstitial membrane and low levels of intra-alveolar edema [32]. In contrast, C3^−/−^ mice showed a partial reduction of respiratory dysfunction, pathology, immune cell infiltration, and cytokine responses in the lung [32]. Additionally, factor B^−/−^ or C4^−/−^ mice also had less weight loss than WT mice [32]. These findings reveal that SARS-CoV infection activates complement systematically and enhances disease exacerbation.

In addition, in a mouse model of infection with the MERS-CoV, the deposition of C5b-9 and C5a both locally and systemically increased after viral infection [33]. Application of C5aR inhibitor attenuated pulmonary inflammation and led to decreased viral replication in infected lung [33].

Taken together, the available data provide evidence that complement is activated in coronavirus infection in experimental models and in patients. In the following section, we will discuss therapeutic anti-complement therapies.

## The therapeutic perspective of complement inhibitors in SARS-CoV-2

As the list of COVID-19-associated pathophysiology linked to deregulated complement activation grows longer, it has become clear that complement inhibitors against C3 or C5 or activating pathways open new windows of therapeutic opportunity for treatment with severe COVID-19 cases. Because they could increase lung oxygenation, reduce lung inflammation, and the systemic complications of ARDS [34]. We provide a review of the complement inhibitors that are currently in clinical trials for COVID-19 (Fig. 1).

### Proximal complement component C3 inhibitor

C3, the central hub of all complement pathways, represents a broad therapeutic target for potential anti-inflammatory therapy in COVID-19. Through inhibition of C3, subsequent production of C3a and C5a and formation of MAC can be prevented. AMY-101, as a highly selective and potent C3 inhibitor, was first successfully used in Italy for the treatment with a COVID-19 patient with severe pneumonia and systemic hyper-inflammation [35–37]. It is currently in Phase II clinical trials for the management of patients with ARDS caused by SARS-CoV-2 infection (ClinicalTrials.gov Identifier: NCT04395456).

### Anti-C5 monoclonal antibody (targeting the terminal pathway)

C5 inhibitors have been used in the clinical practice for almost 15 years, of which the most commonly used is eculizumab, a humanized monoclonal antibody to C5 that prevents cleavage of C5 to C5a and C5b and blocks the formation of terminal complement complex C5b-9. To date, Eculizumab act as one of the complement system inhibitors, it is the only approved drug for FDA (Soliris; Alexion Pharmaceuticals Inc., Cheshire, CT, USA). Eculizumab effectively treats paroxysmal nocturnal hemoglobinuria [37, 38], atypical hemolytic-uremic syndrome [37, 39, 40], transplant-associated thrombotic microangiopathies [36, 41], and autoimmune neuromuscular disorders [42]. Treatment with eculizumab has recently been reported in four severe COVID-19 related ARDS cases in Italy [43]. All patients successfully recovered after the treatment [43]. Based on this, two larger ongoing clinical trials are on the way in Europe (ClinicalTrials.gov Identifier: NCT04355494 and NCT04346797) and the United States (ClinicalTrials.gov Identifier: NCT04288713).

Ravulizumab is a long-acting version of eculizumab that exhibits high-affinity binding to C5 and inhibits C5a and C5b formation. A multicenter Phase 3, open-label randomized, controlled study to evaluate the efficacy and safety of intravenously administered Ravulizumab compared with best supportive care in patients with COVID-19 severe pneumonia, acute lung injury or ARDS is being conducted in acute care hospital settings in the United States, United Kingdom, Spain, France, Germany, and Japan [44]. Yu et al*.* found that C5 monoclonal antibody prevents accumulation of C5b-9 on TF1*PIGA*null cell but not upstream complement activation induced by the SARS-CoV-2 spike proteins [45]. Dense deposition of C3b could cause breakthrough from C5 inhibitors [46, 47]. Therefore, upstream factor H inhibitors might be more specific and effective at preventing the complement-mediated damage in COVID-19 patients [45].

### Anti-C5a monoclonal antibody

The anti-C5a agents aim to selectively block the effect of C5a without affecting the formation of MAC [48, 49]. Thus, targeting C5a might be more effective than targeting C5. In China, two patients with severe COVID-19 benefited from treatment with anti-C5a monoclonal antibody therapy [23]. Multinational randomized controlled trials of the C5a monoclonal antibody, BDB-001 in China (2020L00003) and IFX-1 in Europe (NCT04333420), are already enrolling patients with severe and critical COVID-19 [23, 36, 37].

### Anti-MASP-2 monoclonal antibody (Targeting the LP)

Evidence proved that MASP-2, the lectin pathway’s serine protease, bound with coronavirus N protein and was abnormally highly expressed in lung tissue of COVID-19 patients. Targeting the LP activation can be achieved by applying a human anti-MASP-2 monoclonal antibody [23, 24]. The therapeutic effect of anti-MASP-2 antibody Narsoplimab has been recognized in LP-activated thrombotic microangiopathies [36, 37, 50]. Interestingly, a recent study performed that Narsoplimab improved the clinical inflammation indicators in six COVID-19 patients at the first time, and non-reported adverse drug reactions [51]. However, anti-MASP-2 monoclonal antibody act as a new potential treatment strategy in COVID-19 is needed to confirm in the future.

## Conclusion

Multiple organ involvement has been described in the setting of COVID-19. Although the mechanism is still not clear, emerging clinical and experimental evidence indicate that complement plays a key role in the pathogenesis of COVID-19 and it has become a target for therapeutic intervention. However, the complement system is complicated and involved in a multiphasic role, where inhibiting part of or all three major pathways at the wrong time could potentially lead to harm. Hence, a better understanding of the role of complement in the setting of COVID-19 may help to optimize targeted therapies, including the ideal timing of such therapies in the disease course.
